# Protective effect of *Agrimonia pilosa* polysaccharides on dexamethasone‐treated MC3T3‐E1 cells via Wnt/β‐Catenin pathway

**DOI:** 10.1111/jcmm.14868

**Published:** 2020-01-19

**Authors:** Wei Huang, Shengyang Jin, Wenbo Yang, Shuo Tian, Chunqing Meng, Huan Deng, Hong Wang

**Affiliations:** ^1^ Department of Orthopaedics Union Hospital Tongji Medical College Huazhong University of Science and Technology Wuhan China; ^2^ College of Life Science and Health Wuhan University of Science and Technology Wuhan China

**Keywords:** *Agrimonia pilosa* polysaccharide, apoptosis, steroid‐induced avascular necrosis of the femoral head (SANFH), sulphated polysaccharide, Wnt/β‐Catenin signalling pathway

## Abstract

A water‐soluble polysaccharide (APP‐AW) was isolated from *Agrimonia pilosa* and prepared to three sulphated derivatives (S1, S2 and S3). The results showed that pre‐treatment with APP‐AW, S1, S2 and S3 each at the concentration of 50 μg/mL for 48 hours was able to prevent cytotoxicity induced by 1 μmol/L dexamethasone (Dex) in MC3T3‐E1 cells via inhibition of apoptosis, which is in line with the findings in flow cytometry analysis. Meanwhile, the decreased ALP activity, collagen content, mineralization, BMP2, Runx2, OSX and OCN protein expression in DEX‐treated MC3T3‐E1 cells were reversed by the addition of APP‐AW, S1, S2 and S3. Moreover, APP‐AW, S1, S2 and S3 rescued DEX‐induced increase of Bax, cytochrome c and caspase‐3 and decrease of Bcl‐2, Wnt3, β‐catenin and c‐Myc protein expression in MC3T3‐E1 cells. Our findings suggest that pre‐treatment with APP‐AW, S1, S2 and S3 could significantly protect MC3T3‐E1 cells against Dex‐induced cell injury via inhibiting apoptosis and activating Wnt/β‐Catenin signalling pathway, thus application of these polysaccharides may be a promising alternative strategy for steroid‐induced avascular necrosis of the femoral head (SANFH) therapy.

## 1 INTRODUCTION

Nowadays, long‐term and excessive use of GCs (ie dexamethasone) usually resulted in serious adverse effects including steroid‐induced avascular necrosis of the femoral head (SANFH), which eventually leading to bone mass loss and bone structure deterioration if not remedied in time.^1^, ^2^ Available therapies for SANFH include artificial joint replacement surgery and drug remedies however, most of which are unsatisfactory.^3^ Indeed, most patients with SANFH eventually are subjected to total hip arthroplasty within a few years.^4^ Although the aetiology and pathogenesis of SANFH remains controversial, it has been widely accepted that the imbalance between osteoblastic bone formation and osteoclastic bone resorption results in excessive bone loss, leading to various chronic bone diseases including SANFH.^5^ It was suggested that apoptosis and differentiation of bone cell has an important role in the initiation and pathogenesis of femoral head necrosis.^6^, ^7^ Numerous in vitro and in vivo studies also strongly suggested that GCs were able to inhibit the growth and differentiation of bone cells, thus affecting the reconstruction and resorption of bones and decreasing the bone conversion rate, which further leads to bone destruction, and the deposit of apoptotic bone cells.^8^ Therefore, it is critical to understand the pathological mechanisms underpinning this condition and to develop proper therapeutics to disrupt the progression of SANFH.

In the past few years, a large body of evidence suggests that natural‐product‐based traditional Chinese drugs have attracted more and more attention for treating bone diseases with less toxicity and more efficiency.^9^, ^10^ In our previous study, we isolated one homogenous polysaccharide APP‐AW from the dried aerial parts of *Agrimonia pilosa* and it was identified to be β‐(1 → 3)‐d‐glucan, based on a combination of chemical and instrumental analysis. The effect of APP‐AW on Dex‐induced apoptosis in primary murine osteoblaststs was also examined. The results showed that exposure of APP‐AW significantly attenuated cell loss induced by Dex in osteoblasts via inhibition of apoptosis.^11^ As on ongoing work for expanding use of this polysaccharide in the treatment of SANFH, the main objective of the present study was to examine the effect of APP‐AW and its sulphated derives on the cell proliferation and differentiation of MC3T3‐E1 cells induced by Dex. Furthermore, the possible molecular mechanism underlying was investigated in the present study.

## 2 MATERIALS AND METHODS

### 2.1 Materials and chemicals

*Agrimonia pilosa* was purchased from the local Drug store in Wuhan city of China. Dulbecco's Modified Eagle's medium (DMEM) and foetal bovine serum (FBS) were purchased from Gibco/Invitrogen. Annexin V‐FITC/propidium iodide (PI) apoptosis detection kit was from Keygentec. Dexamethasone (Dex) and Cell Counting Kit‐8 (CCK‐8) was obtained from Beyotime Institute of Biotechnology. BCA protein assay kit and ALP Assay kit was from Nanjing Jiancheng Bioengineering Research Institute. Antibodies to Bax, Bcl‐2, cytochrome c, Runx2, caspase‐3, osterix (OSX), osteocalcin (OCN), BMP‐2, Wnt3, β‐catenin, c‐Myc and β‐actin were purchased from Santa Cruz Biotechnology, Inc. All other chemical reagents used in this experiment were of analytical grade purchased from Sigma Chemical Co.

### 2.2 Preparation of polysaccharide AAP‐AW and its sulphated derives

The purified polysaccharide, APP‐AW, was isolated from *A pilosa* as described in the early published work.^11^ Sulphated modification of polysaccharide (AAP‐AWS1, AAP‐AWS2 and APP‐AWS3) was carried out using the chlorosulphonic acid‐pyridine (CSA–Pyr) method.^12^ A calibration curve was constructed with sodium sulphate as standard. The sulphate group content in polysaccharides was determined by the BaCl_2_–gelatin method of Kawai et al.^13^

### 2.3 Cells culture

Mouse pre‐osteoblastic MC3T3‐E1 cells were purchased from China Center for Type Culture Collection (CCTCC) (Wuhan China) and grown in DMEM supplemented with 10% FBS, penicillin (100 U/mL) and streptomycin (100 μg/mL) at 37°C in a humidified incubator containing 5% CO_2_. For the induction of osteoblast differentiation, MC3T3‐E1 cells were cultured in DMEM containing 10% FBS, penicillin (100 U/mL), streptomycin (100 μg/mL), l‐ascorbic acid (50 μg/mL) and β‐glycerophosphate disodium salt hydrate (10 mmol/L) under the same conditions and then treated with 1 μmol/L DEX or vehicle control. The medium was changed every 3 days.

### 2.4 Cell viability assay

Cell viability was measured with CCK‐8 assay as described previously.^14^ Briefly, MC3T3‐E1 cells were seeded in 96‐well plates at the density of 5 × 10^3^ cells/mL at 37°C in humidity incubator with 5% CO_2_ until 80% confluence was reached. To assay the effect of APP‐AW or DEX alone on osteoblast viability, the cells were treated with various concentrations of test sample (0, 5, 10, 25, 50 and 100 μg/mL) for 24, 48 and 72 hours, or DEX (0.25, 0.5, 1, 2 and 5 μmol/L) for 24 hours. For assessment of the effect of AAP‐AW, S1, S2 and S3 pre‐treatment on DEX‐induced osteoblast viability, cells were first pre‐treated with or without 10, 25 and 50 μg/mL of test sample for 48 hours and then treated 1 μmol/L DEX for another 24 hours. Subsequently, 10 μL of CCK‐8 solution was added to each well and the wells were cultured for an additional 2 hours. After shaking for 1 minute, the optical density (OD) was measured at 450 nm using a microplate reader (Bio‐Rad). The ratio of the mean OD between treated and control group was considered to reflect the relative cellular viability. The experiment was performed in triplicate.

### 2.5 Annexin V‐FITC/PI staining assay

Cell apoptosis was assessed by double staining with Annexin V‐fluorescein isothiocyanate (FITC) and propidium iodide (PI) followed by flow cytometry analysis as described previously.^15^ The cells stained with 5 μL Annexin V‐FITC (10 μg/mL) and 5 μL propidium iodide (PI, 5 μg/mL) were immediately analysed on a FACScan flow cytometry using Cell‐Quest software (Becton Dickinson). In each analysis, 10 000 events were recorded.

### 2.6 ALP activity

To examine the ALP activity in MC3T3‐E1 cells subjected to the different treatments, the cells were washed twice, harvested and resuspended in 100 μL of lysis buffer (0.2% Triton X‐100) after 48 hours of culture. Then, the supernatant from lysate by centrifugation (12 000*g*, 5 minutes) was harvested for the assay of ALP activity (U/mL) with an ALP Assay kit according to the manufacturer's protocol. All samples were examined in triplicate.

### 2.7 Measurement of collagen content

MC3T3‐E1 cells were seeded in 12‐well plates at a density of 5 × 10^5^ cells/well for 24 hours. When the cells reach 80% confluence, the cells were treated with or without samples (10, 25 and 50 μg/mL) for 48 hours, followed by the addition of 1 μmol/L DEX for another 24 hours in the presence of samples or not. After the exposure period, the cells were harvested, washed, fixing with Bouin's fluid for 1 and then stained with 100 µL of 0.1% (wt/vol) Sirius Red dye for 1 hour at 37°C. After being washed with 200 µL of HCl solution (10 mmol/L) for three times, the plated was added with 0.1 mol/L NaOH to dissolve the adsorbed collagen for 30 minutes. The absorbance was measured on a microplate reader at a wavelength of 550 nm and the relative collagen content was calculated using the following formula: absorbance of treated cells/absorbance of untreated cells × 100%.

### 2.8 Alizarin Red S staining

Mineralized nodules in the MC3T3‐E1 cells were determined by staining with alizarin red S, which selectively binds to calcium and yields a dark red stain. Pre‐osteoblast MC3T3‐E1 cells were cultured in 6‐well plates with osteogenic differentiation medium. Following incubation with or without samples at different concentrations of 10, 25 and 50 μg/mL for 48 hours, MC3T3‐E1 cells were incubated with 1 μmol/L DEX for another 24 hours in the presence of samples or not. Thereafter, cells were cultured only with osteogenic differentiation medium, which was replenished every 3 days. After osteogenic induction for 15 days, the cells were rinsed 3 times with PBS, fixed with 4% paraformaldehyde for 30 minutes at 4°C, then washed twice with distilled water and stained with 40 mmol/L alizarin red S at room temperature for 5 minutes while being gently swayed. Mineralized nodules were photographed under a light microscope (CKX31; Olympus) and quantified by the number of calcified nodules formed per unit area.

### 2.9 Protein extraction and Western blot analysis

Equal proteins of each sample (30 μg) were resolved by 12% sodium dodecyl sulphate‐polyacrylamide gel electrophoresis (SDS‐PAGE) and electrophoretically transferred onto polyvinylidene difluoride (PVDF) membranes. The membranes were blocked with 5% fat‐free milk and then incubated with primary antibodies specific to Bax, Bcl‐2, cytochrome c, Runx2, caspase‐3, OSX, OCN, BMP‐2, Wnt3, β‐catenin, c‐Myc and β‐actin at 1:1000 dilution overnight at 4°C, followed by hybridization with horseradish peroxidase‑conjugated secondary antibody (1:1000) for 1 hour at room temperature. After that, the membranes were washed thrice with TBST and visualized using an enhanced chemiluminescence kit. The band density was quantified using Image J software and the relative protein levels normalized to that of β‐actin.

### 2.10 Statistical analysis

The data are presented as means ± SD The differences between the groups were analysed with analysis of variance (ANOVA). *P*‐values of less than .05 were accepted to be statistically significant.

## 3 RESULTS

### 3.1 Characterization of AAP‐AW sulphated derives

Three sulphated derivatives of AAP‐AW (S1, S2 and S3) were prepared by CSA–Pyr method. Total carbohydrate content, Mw and DS of the sulphated derivatives were listed in Table 1. The DS of the sulphated derivatives were listed in an increasing order as follows: S1 < S2 < S3. In contrast, the carbohydrate contents of them are in an opposite order: S1 > S2 > S3. They all showed a single and symmetrical peak on HPSEC (data not shown), and the average molecular weights of S1, S2 and S3 were about 11.80, 13.22 and 15.78 kD, respectively, in reference to the standard curve made from T‐Dextran.

**Table 1:** Characterization of the sulphated AAP‐AW (S1, S2 and S3)

Samples	CSA:Pyr	Carbohydrate content (%)	Mw (kD)	DS
S1	1:4	76.38	11.80	0.90
S2	1:2	58.54	13.22	1.51
S3	1:1	46.12	15.78	2.15

### 3.2 AAP‐AW and its sulphated derives inhibit DEX‐induced cytotoxicity in MC3T3‐E1 cells

**Figure 1:**
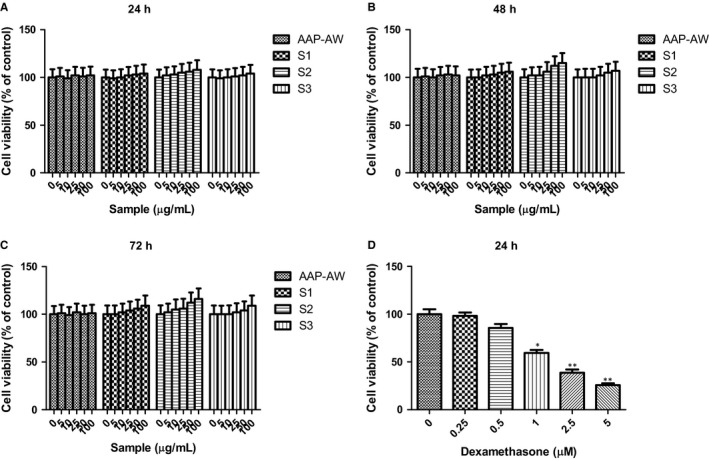
(A) Effects of AAP‐AW, S1, S2 and S3 on the cell viability of MC3T3‐E1 cells at 24 h; (B) effects of AAP‐AW, S1, S2 and S3 on the cell viability of MC3T3‐E1 cells at 48 h; (C) effects of AAP‐AW, S1, S2 and S3 on the cell viability of MC3T3‐E1 cells at 72 h; (D) effects of dexamethasone (DEX) on the cell viability of MC3T3‐E1 cells at 24 h. Data are presented as the mean ± SD. *P < .05, **P < .01 vs normal control

**Figure 2:**
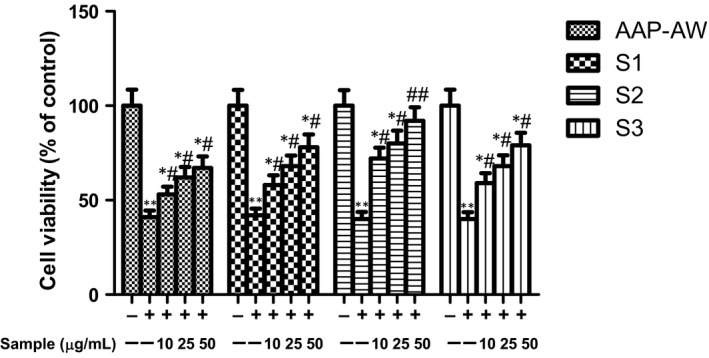
Effects of AAP‐AW, S1, S2 and S3 pre‐treatment for 48 h on the cell viability of Dex (1 μmol/L)‐treated MC3T3‐E1 cells in vitro. Data are presented as the mean ± SD. *P < .05, **P < .01 vs control; #*P* < .05, ##*P* < .01 vs DEX control

As shown in Figure 1A‐D, AAP‐AW almost showed not any inhibitory or promoting effect on cell growth of MC3T3‐E1 cells, whereas treatment of MC3T3‐E1 cells with S1, S2 and S3 at concentrations ranging from 0 to 100 μg/mL with the passage of time marginally increased the cell growth in a concentration and time‐dependent manner with an decreasing order: S2 > S3 > S1 > AAP‐AW at each time point. As well, the cells that were treated with DEX displayed decreasing cell survival rate at 24 hours, especially beyond 1 μmol/L as compared with untreated control (*P* < .05 or *P* < .01). Intriguingly, incubation with AAP‐AW, S1, S2 and S3 for 48 hours prior to DEX (1 μmol/L) exposure for 24 hours significantly increased the cell viability of MC3T3‐E1 cells in the same order as above (Figure 2). Of note, the high dose of S2 at 50 μg/mL exhibited the maximum potency against DEX‐induced cell death in MC3T3‐E1 cells.

### 3.3 AAP‐AW and its sulphated derives attenuate DEX‐induced apoptosis in MC3T3‐E1 cells

**Figure 3:**
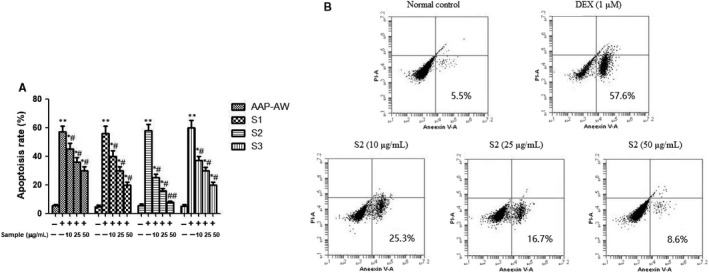
(A) Effects of AAP‐AW, S1, S2 and S3 on Dex (1 μmol/L)‑induced apoptosis in MC3T3‐E1 cells. (B) Representative figure of anti‐apoptotic effect of S2 in MC3T3‐E1 cells by flow cytometric analysis. Data are presented as the mean ± SD. *P < .05, **P < .01 vs control; #*P* < .05, ##*P* < .01 vs DEX control

The percentage of apoptosis in different group was measured by Annexin V‑FITC/PI flow cytometric analysis (Figure 3A,B). Exposure of MC3T3‐E1 cells to 1 μmol/L DEX for 24 hours markedly induced cell apoptosis, while pre‐treatment with AAP‐AW, S1, S2 and S3 for 48 hours significantly attenuated this effect in a dose‐dependent manner and behaved in the same order as for cell survival, namely S2 > S3 > S1 > AAP‐AW. Next, we would only choose 50 μg/mL of AAP‐AW, S1, S2 and S3 as research targets for following assays.

### 3.4 AAP‐AW and its sulphated derives improves DEX‐induced dysfunction of the ALP activity, collagen content and mineralization in MC3T3‐E1 cells

**Figure 4:**
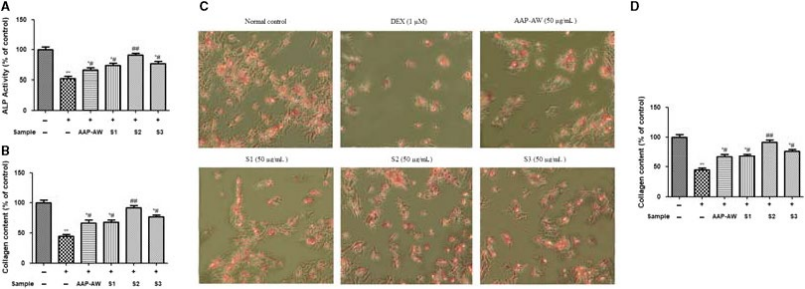
(A) Effects of AAP‐AW, S1, S2 and S3 on ALP activity of Dex (1 μmol/L)‐treated MC3T3‐E1 cells in vitro. (B) Effects of AAP‐AW, S1, S2 and S3 on collagen content of Dex (1 μmol/L)‐treated MC3T3‐E1 cells in vitro. (C) Representative figure of Alizarin Red staining of the MC3T3‐E1 cells with different treatment. (D) Effects of AAP‐AW, S1, S2, and S3 on mineralization of Dex (1 μmol/L)‐treated MC3T3‐E1 cells in vitro. Data are presented as the mean ± SD. *P < .05, **P < .01 vs control; #*P* < .05, ##*P* < .01 vs DEX control

ALP activity showed significant differences between treatment with DEX and polysaccharide samples, alone or combined (Figure 4A). The MC3T3‐E1 cells in the DEX group displayed less ALP activity than those of the control group (*P* < .01), while ALP activity in AAP‐AW, S1, S2 and S3 group was significantly increased at the concentration of 50 μg/mL, especially for S2, when compared with the DEX‑treated group (*P* < .05 or *P* < .01).

As seen in Figure 4B, the ability of DEX, AAP‐AW and its sulphated derives to synthesize collagen in the MC 3T3‐E1cells was similar to their effects on ALP activity. The decreased collagen secretion induced by DEX was significantly rescued in MC 3T3‐E1 cells after treatment with 50 μg/mL of AAP‐AW, S1, S2 and S3 for 48 hours. The maximal collagen content in MC3T3‐E1 cells was observed in cells treated with 50 μg/mL of S2.

As shown in Figure 4C, the calcified nodules appeared red in colour by Alizarin Red S staining. In line with the results on ALP activity and collagen synthesis, the quantitative analysis result of Alizarin Red S staining, as showed in Figure 4D, indicated that the number of calcified nodules was decreased with the addition of DEX, whereas the presence of AAP‐AW, S1, S2 and S3 at a concentration of 50 μg/mL showed significant enhancing effect on mineralization compared with that in the control group (*P* < .05 or *P* < .01). The number of calcified nodules reached its maximum when cells were treated with 50 μg/mL of S2 in osteogenic differentiation medium for 15 days.

### 3.5 AAP‐AW and its sulphated derives regulate DEX‐induced osteoblast‐specific protein expression in MC3T3‐E1 cells

**Figure 5:**
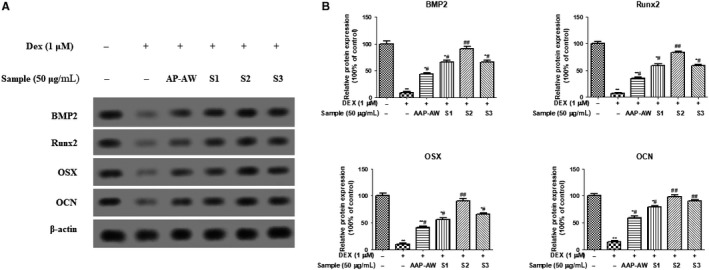
(A) Effects of AAP‐AW, AAP‐AWS1, AAP‐AWS2, and AAP‐AWS3 on the protein expression of BMP2, Runx2, OSX and OCN of Dex (1 μmol/L)‐treated MC3T3‐E1 cells in vitro. (B) The relative protein expression levels. Data are presented as the mean ± SD. *P < .05, **P < .01 vs control; #*P* < .05, ##*P* < .01 vs DEX control

The Western blot assay revealed that, following incubation, the MC3T3‐E1 cells in the DEX group displayed less protein expression of BMP2, Runx2, OSX and OCN than those of the control group (*P* < .01), while the MC3T3‐E1 cells treated with AAP‐AW, S1, S2 and S3 exhibited an increased expression for these proteins, particularly the cells treated with 50 μg/mL of S2 (Figure 5).

### 3.6 AAP‐AW and its sulphated derives regulate DEX‐induced apoptosis protein expression in MC3T3‐E1 cells

**Figure 6:**
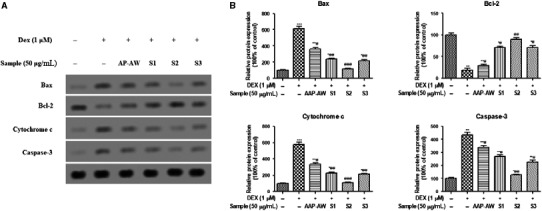
(A) Effects of AAP‐AW, S1, S2, and S3 on the protein expression of Bax, Bcl‐2, cytochrome c, and caspase‐3 of Dex (1 μmol/L)‐treated MC3T3‐E1 cells in vitro. (B) The relative protein expression levels. Data are presented as the mean ± SD. *P < .05, **P < .01, ***P < .001 vs control; #*P* < .05, ##*P* < .01, ###*P* < .001 vs DEX control

**Figure 7:**
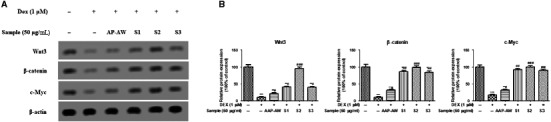
(A) Effects of AAP‐AW, S1, S2, and S3 on the protein expression of Wnt3, β‐catenin and c‐Myc of Dex (1 μmol/L)‐treated MC3T3‐E1 cells in vitro. (B) The relative protein expression levels. Data are presented as the mean ± SD. *P < .05, **P < .01, ***P < .001 vs control; #*P* < .05, ##*P* < .01, ###*P* < .001 vs DEX control

As shown in Figure 6, compared with normal control cells, the protein expression of Bax cytochrome c and caspase‐3 was significantly elevated following Dex (1 μmol/L) alone treatment (*P* < .01 or *P* < .001). Simultaneously, treatment with Dex for 48 hours obviously led to the decreased protein expression of Bcl‐2. On the contrary, pre‐treatment with AAP‐AW, S1, S2 and S3 showed the opposite trend and reach the highest value for S2 at the concentration of 50 μg/mL (Figure 7).

### 3.7 AAP‐AW and its sulphated derives regulate DEX‐induced Wnt/β‐catenin signalling protein expression in MC3T3‐E1 cells

To clarify if the involvement of Wnt/β‐catenin signalling in the protective effect of AAP‐AW and its sulphated derives against DEX‐induced apoptosis in MC3T3‐E1 cells, MC3T3‐E1 cells exposed to different treatment as described before were subject to protein examination of Wnt3, β‐catenin and c‐Myc. It was clear that significant decreased protein expression of Wnt3, β‐catenin and c‐Myc was observed in DEX (1 μmol/L)‐treated MC3T3‐E1 cells compared with the normal cells (*P* < .001), while the addition of AAP‐AW, S1, S2 and S3 resulted in an increase of these protein expressions.

## 4 DISCUSSION

Steroid‐induced avascular necrosis of the femoral head is a common orthopaedic disease with difficult rehabilitation and poor prognosis, threatening the health and quality of normal life of patients.^16^ Accumulating evidence suggests that long‐term or excessive GCs use is the most common non‐traumatic cause of SANFH.^2^ Owing the complexity of the pathogenesis of SANFH, new therapy approaches for this disease have been extensively investigated.

In this study, to elucidate the role of AAP‐AW and its sulphated derives in bone formation and growth; the cultured MC3T3‐E1 cells suffering Dex exposure were created to a cellular model of SANFH. The results of MTT assay indicated that no cell viability change in AAP‐AW‐treated cells when compared with normal cells. Its three sulphated derives (S1, S2 and S3) marginally increased the cell growth of MC3T3‐E1 in a time and concentration‐dependent manner, although the effect was not significant from the normal cells. Apparently, the maximum value was observed in cells treated with S2. Also, exposure of MC3T3‐E1 cells to DEX beyond 1 μmol/L for 24 hours led to dramatic cytotoxicity, evidenced by the decreased cell viability. More importantly, the addition of AAP‐AW, S1, S2 and S3 greatly reversed the cell loss induced by DEX (1 μmol/L) in MC3T3‐E1 cells, and S2 showed the maximum protective effect. This trend was in line with the observations in cell apoptosis assay. Flow cytometry analysis indicated that only a small amount of cells survived in MC3T3‐E1 cells after being treated with DEX in FBS‐free medium for 24 hours, while pre‐treatment with AAP‐AW, S1, S2 and S3 for 48 hours significantly decreased apoptotic rate of DEX‐treated MC3T3‐E1 cells, with lowest apoptosis rate in S2 group.

ALP activity and collagen synthesis are two important phenotypic markers for osteoblastogenesis in the early‐stage, directly reflecting osteoblast activity and/or function.^17^, ^18^ They are associated with matrix maturation and mineralization in the terminal stage of bone formation.^19^ To verify whether AAP‐AW and its sulphated derives can enhance the osteogenic differentiation potential of DEX‐treated MC3T3‐E1 cells, both early (the ALP activity, collagen content) and mature osteogenic markers (mineralization) were assayed. Compared with the control group, ALP activity was significantly reduced in DEX group, while the MC3T3‐E1 cells in the other groups exhibited an increased ALP level, particularly in the cells treated with S2. Meanwhile, the collagen content was increased in MC3T3‐E1 cells for incubation with AAP‐AW, S1, S2 and S3 for 48 hours that displayed statistically significant from DEX control group and the collagen content was higher in group treated with 50 μg/mL of S2 than others. As expected, the similar effect was also verified on the mineralization of MC3T3‐E1 cells. These findings indicated that AAP‐AW and its sulphated derives could cause osteoblastogenic differentiation and accumulation of collagen, and finally leads to calcium deposition in MC3T3‐E1 cells exposed to DEX treatment.

Runx2 is a master transcription factor in the early‐stage of osteoblast differentiation and is closely correlated with the expression of osteoblast marker genes and related proteins in the late‐stage molecular events of osteoblast differentiation, such as OSX and OCN.^20^ While, as a key signalling member of the TGF‑β super family, BMP‐2 is indispensable for osteoblast proliferation and osteogenic differentiation and accounts for osteogenic effect of osteoblasts to a great extent.^21^, ^22^ Furthermore, the changes in the expression of Runx2, BMP‐2, OSX and OCN were also investigated in MC3T3‐E1 cells in response to different treatment. We observed that cells treated with 1 μmol/L DEX exhibited decreased protein expression of BMP‐2, Runx2, OSX and OCN compared with the normal control. However, AAP‐AW, S1, S2 and S3 corrected DEX‐induced decrease in these proteins expression, particularly with 50 μg/mL of AAP‐AWS2. These data demonstrated that AAP‐AW and its sulphated derives, especially S2, can enhance the osteogenic differentiation protein expression in MC3T3‐E1 cells.

Apart from improvement of AAP‐AW and its sulphated derives on the osteogenic differentiation at the molecular level, DEX‐induced osteoblast apoptosis significantly contributes to the development of SANFH and this programmed cell death is regulated by a variety of genes, including Bax, Bcl‐2, cytochrome c, caspase‐3 and so on.^15^ The western blot results indicated that pre‐treatment with AAP‐AW, S1, S2 and S3 significantly reversed the downregulated protein expression of Bcl‐2 and upregulated protein expression of Bax, cytochrome c and caspase‐3 in MC3T3‐E1 cells induced by DEX, which is in line with the results of Annexin V‐FITC/PI flow cytometry analysis. This result suggested that the protective effect of pre‐treatment with AAP‐AW and its sulphated derives against DEX‐induced osteoblast apoptosis was mediated by the inhibition of the mitochondrial apoptosis pathway.

There is abundant evidence suggesting that the Wnt/β‐catenin signalling pathway was involved in the pathogenesis of early‐stage ANFH by regulating osteoblast differentiation and bone formation.^15^ The downstream genes of the Wnt/β‐catenin pathway, such as Bcl‐2 and c‐myc, are responsible for the cell cycle and cell apoptosis.^23^ Several lines of evidence indicate that DEX‐induced inhibition of Wnt/β‐cateni pathway increased the rate of apoptosis of osteoblasts in vitro or in vivo.^24^ Similarly, the decreased protein expression of Wnt3, β‐catenin and c‐Myc was also observed in MC3T3‐E1 cells exposed to DEX (1 μmol/L), whereas this suppression was restored by the pre‐treatment with AAP‐AW, S1, S2 and S3 and reached the higher degree in S2 group than others. Another indispensable finding in the present study was that Wnt/β‐catenin signalling pathway was involved in the protective mechanism by which AAP‐AW and its sulphated derives regulated cell growth of osteoblasts.

## 5 CONCLUSIONS

In conclusion, the present study had for the first time demonstrated that AAP‐AW and its sulphated derives enhanced osteogenic osteoblast proliferation and osteogenic differentiation under conditions of DEX‐induced dysfunction of MC3T3‐E1 cells in vitro via Wnt/β‐catenin signalling pathway through maintaining cellular survival, promoting osteogenic differentiation and inhibiting cell apoptosis. These results indicated that pre‐treatment with AAP‐AW and its sulphated derives may be a promising therapeutic strategy for the prevention and treatment of SANFH. To fully elucidate the mechanism involved, more well‐designed studies are required in future.

## CONFLICT OF INTEREST

The authors declare that they have no competing interests.

## AUTHOR CONTRIBUTIONS

WH and HW designed the experiment and interpreted results. WH and HD drafted manuscript. WH, SYJ, WBY, ST, CQM and HD performed experiments. All authors have read and approved the final manuscript. HD and WH are co‐corresponding authors.

## Data Availability

All data used to support the findings of this study are available from the corresponding authors on reasonable request.
